# Comprehensive deciphering prophages in genus *Acetobacter* on the ecology, genomic features, toxin–antitoxin system, and linkage with CRISPR-Cas system

**DOI:** 10.3389/fmicb.2022.951030

**Published:** 2022-08-02

**Authors:** Chenggong Qian, Jiawen Ma, Jiale Liang, Lei Zhang, Xinle Liang

**Affiliations:** School of Food Science and Biotechnology, Zhejiang Gongshang University, Hangzhou, China

**Keywords:** *Acetobacter*, prophage, co-evolution, vinegar, genomic analysis

## Abstract

*Acetobacter* is the predominant microbe in vinegar production, particularly in those natural fermentations that are achieved by complex microbial communities. Co-evolution of prophages with *Acetobacter*, including integration, release, and dissemination, heavily affects the genome stability and production performance of industrial strains. However, little has been discussed yet about prophages in *Acetobacter*. Here, prophage prediction analysis using 148 available genomes from 34 *Acetobacter* species was carried out. In addition, the type II toxin–antitoxin systems (TAs) and CRISPR-Cas systems encoded by prophages or the chromosome were analyzed. Totally, 12,000 prophage fragments were found, of which 350 putatively active prophages were identified in 86.5% of the selected genomes. Most of the active prophages (83.4%) belonged to the order *Caudovirales* dominated by the families *Siphoviridae* and *Myroviridae* prophages (71.4%). Notably, *Acetobacter* strains survived in complex environments that frequently carried multiple prophages compared with that in restricted habits. *Acetobacter* prophages showed high genome diversity and horizontal gene transfer across different bacterial species by genomic feature characterization, average nucleotide identity (ANI), and gene structure visualization analyses. About 31.14% of prophages carry type II TAS, suggesting its important role in addiction, bacterial defense, and growth-associated bioprocesses to prophages and hosts. Intriguingly, the genes coding for Cse1, Cse2, Cse3, Cse4, and Cas5e involved in type I-E and Csy4 involved in type I-F CRISPR arrays were firstly found in two prophages. Type II-C CRISPR-Cas system existed only in *Acetobacter aceti*, while the other *Acetobacter* species harbored the intact or eroded type I CRISPR-Cas systems. Totally, the results of this study provide fundamental clues for future studies on the role of prophages in the cell physiology and environmental behavior of *Acetobacter*.

## Introduction

Acetic acid bacteria (AAB), a group of Gram-negative and aerobic bacilli that can be isolated from flowers, fruits, soil, intestines, wine, and vinegar, are currently classified into 19 genera (Qiu et al., 2021). The *Acetobacter, Gluconobacter*, and *Komagataeibacter* species have been widely studied for their ability to efficiently oxidize ethanol to vinegar (Yang et al., 2022). However, abnormal vinegar fermentation is often observed, and the underlying mechanism remains unclear due to the complicated microbial community combined with uneven fermentation technology. The multi-omics studies on traditional food fermentations have indicated the impacts conferred by the bacterial community fluctuation and the environmental conditions on the fermentation process (Das and Tamang, 2021; Ma et al., 2022; Yang et al., 2022; Zotta et al., 2022). In parallel, the recent findings of virome contributions to the evolution of bacterial communities rely on the ocean, soil, and gut metagenome investigations, which suggest the potential of phages in shaping the architecture and development of fermented food communities (Roy et al., 2020; Chevallereau et al., 2022; Ge et al., 2022). Several phages have been adopted and engineered as alternative antimicrobials or therapeutic potency successfully (Bao et al., 2020; Ge et al., 2021, 2022; Grabowski et al., 2021). Phageomes in the cheese starters and fermented vegetables were determined, and virulent and temperate phages were isolated and sequenced. Recently, the analysis of historical and experimental samples from 82 years of Swiss hard cheese starter culture propagation showed that strains differ tremendously in their phage resistance capacity (Somerville et al., 2022). The co-evolution of phages and bacteria together has addressed the driver force of the community balance (Mancini et al., 2021; Harvey and Holmes, 2022).

Prophages are potential phages that integrate their genes into the host chromosomes after the invasion and replicate simultaneously with the host. Under physiological stress conditions, prophage can enter into the lytic stage, resulting in the death of a lysogenic host cell. This kind of switching helps to tune the dynamic balance of populations in the development of the bacterial community (Secor and Dandekar, 2020). For example, prophages co-evolved with *Lactobacillus plantarum* and helped the host to survive the acidic kimchi fermentation (Park et al., 2022). The temperate phage ΦAP1.1 exhibited a recurrent mechanism to counteract bacterial immunity by inserting itself into the CRISPR repeats and thus neutralizing the *Streptococcus pyogenes* immunity (Varble et al., 2021). In addition, prophages could promote horizontal gene transfer (HGT) among bacterial populations (Magaziner et al., 2019). The auxiliary gene modules confer the properties of virulence and antibiotic resistance through transduction pathways to the host (Naorem et al., 2021; Wendling et al., 2021). Meanwhile, several anti-CRISPR (Acr)-encoded genes in prophage have been found as a defense approach in the arms race with hosts (Wang et al., 2020), while genes encoding for hydrolysis enzymes were involved in the recycling of whole ocean materials. Currently, eight types of toxin–antitoxin systems (TAs) have been uncovered and annotated (Alvarez et al., 2020; Jurenas et al., 2022). Also, TAs on prophages are involved in prophage induction and regulation of the physiological state of cells (Li et al., 2020; Song and Wood, 2020; Jurenas et al., 2021). Previous studies suggested that diverse type II TAs are present in the active prophages of *Acetobacter* genomes (Omata et al., 2021; Xia et al., 2021b). Nevertheless, a full investigation of the distribution of prophages in the genus *Acetobacter* has not been performed yet.

Actually, several cases of contamination due to phages during the vinegar fermentation process were reported, and four phages were isolated (Schocher et al., 1979; Stamm et al., 1989; Kiesel and Wünsche, 1993; Kharina et al., 2015). In addition, a *Tectivirus-*infecting *Gluconobacter cerinus* was isolated from wine musts (Philippe et al., 2018), and six temperate phages were obtained from *Acetobacter pasteurianus* (Omata et al., 2021). Furthermore, the available genomic data exhibit plenty of putative prophage loci in *Acetobacter* genomes, as well as in transposons, insertion sequences (ISs), and phage-associated genes, which are associated with some evolutionary and genetic advantages but also contribute to unstable genome features in *Acetobacter* (Yang et al., 2022). Recently, the available genomic data provide us a chance to comprehensively decipher the profile of *Acetobacter* prophage and its important role in physiology, evolution, and genetic diversity of *Acetobacter*. In this study, we aimed to characterize the prophage profiles of *Acetobacter* based on 148 genomes obtained from 34 *Acetobacter* species, and tried to decipher the internal relations of ecology, genomic features, TA distribution, and CRISPR-Cas systems.

## Materials and methods

### *Acetobacter* genome collection

*Acetobacter* genomes (148) were obtained from the National Center for Biotechnology Information (NCBI) (https://www.ncbi.nlm.nih.gov/genome/?term=Acetobacter) database. These genomes represent 34 different species, including *Acetobacter aceti* (9), *Acetobacter ascendens* (3), *Acetobacter cerevisiae* (3), *Acetobacter cibinongensis* (3), *Acetobacter conturbans* (1), *Acetobacter estunensis* (2), *Acetobacter fabarum* (3), *Acetobacter fallax* (2), *Acetobacter farinalis* (1), *Acetobacter ghanensis* (2), *Acetobacter indonesiensis* (4), *Acetobacter lambici* (1), *Acetobacter lovaniensis* (2), *Acetobacter malorum* (7), *Acetobacter musti* (1), *Acetobacter nitrogenifige* (2), *Acetobacter oeni* (3), *Acetobacter okinawensis* (4), *Acetobacter orientalis* (7), *Acetobacter orleanensis* (4), *Acetobacter oryzifermentans* (2), *Acetobacter oryzoeni* (1), *Acetobacter papayae* (1), *Acetobacter pasteurianus* (32), *Acetobacter peroxydans* (3), *Acetobacter persici* (7), *Acetobacter pomorum* (8), *Acetobacter sacchari* (1), *Acetobacter senegalensis* (4), *Acetobactersicerae* (1), *Acetobacter suratthaniensis* (1), *Acetobacter syzygii* (7), *Acetobacter thailandicus* (5), and *Acetobacter tropicalis* (11). The detailed information on these strains is listed in Supplementary Table S1.

### Prophage prediction, ANI analysis, and genome annotation

Prophage Hunter was adopted as the guide for finding prophages. By measuring genomic similarity at the nucleotide level, the putative region with a score of more than or equal to 0.8 was defined as an active prophage, the region with a score between 0.8 and 0.5 as an unspecified prophage, and the region with a score of <0.5 as an inactive prophage (Song et al., 2019). The detailed information is listed in Supplementary Table S2. FastANI v1.3 was used to calculate the ANI value of prophages (Jain et al., 2018). Compared with prophage itself, the derived ANI value was recorded as 100%, and the default ANI value <70% was recorded as not accessible (NA). To annotate the prophage genome region precisely, the Rapid Annotation Using Subsystem Technology (RAST) software was applied (Aziz et al., 2008).

### Prediction, analysis of type II toxin–antitoxin system, and CRISPR-Cas system

TAfinder tool on the TADB website (https://bioinfo-mml.sjtu.edu.cn/TADB2/tools.html) was used to search for type II TA loci (Xie et al., 2018). E-value for BLAST was set at 0.01, E-value for HMMer was set at 1, the maximum length of potential toxin/antitoxin was set at 300 amino acids, and the maximum distance (or overlap) between the potential toxin and antitoxin was set at - 20_ 150 nt. CRISPRCasFinder (https://crisprcas.i2bc.parissaclay.fr/CrisprCasFinder/Index) was then used to find CRISPR alignments, direct repeats, and spacers. According to the guidelines, only levels 3 and 4 CRISPR arrays were counted in this study. Spacer-prophage matching was performed using a local BLAST search, and the following parameters were adopted: blastn-short comparison, query-NA, E-value <1e-5, and outfmt at 6 (Abby et al., 2014; Couvin et al., 2018).

### Data analysis and visualization

The Kruskal–Wallis test and the Mann–Whitney U-test were performed using SPSS PASW Statistics v18.0. Data aggregation and processing were done using Microsoft Office Excel 2016 with the assistance of Python. Point and line plots, bar graphs, box plots, violin plots, and pie charts were visualized using Origin 2021. Strain biogenesis geographic distribution maps and genomic CDS maps were visualized using R. Heatmap visualization and hierarchical clustering were done using HemI (Heatmap Illustrator v1.0) (Deng et al., 2014). The network relationship diagram was visualized using Cytoscape (Shannon et al., 2003). All figures were further embellished and retouched using Adobe Illustrator 2020.

## Results

### Ecological distribution of prophage diversity in *Acetobacter*

**Figure 1:**
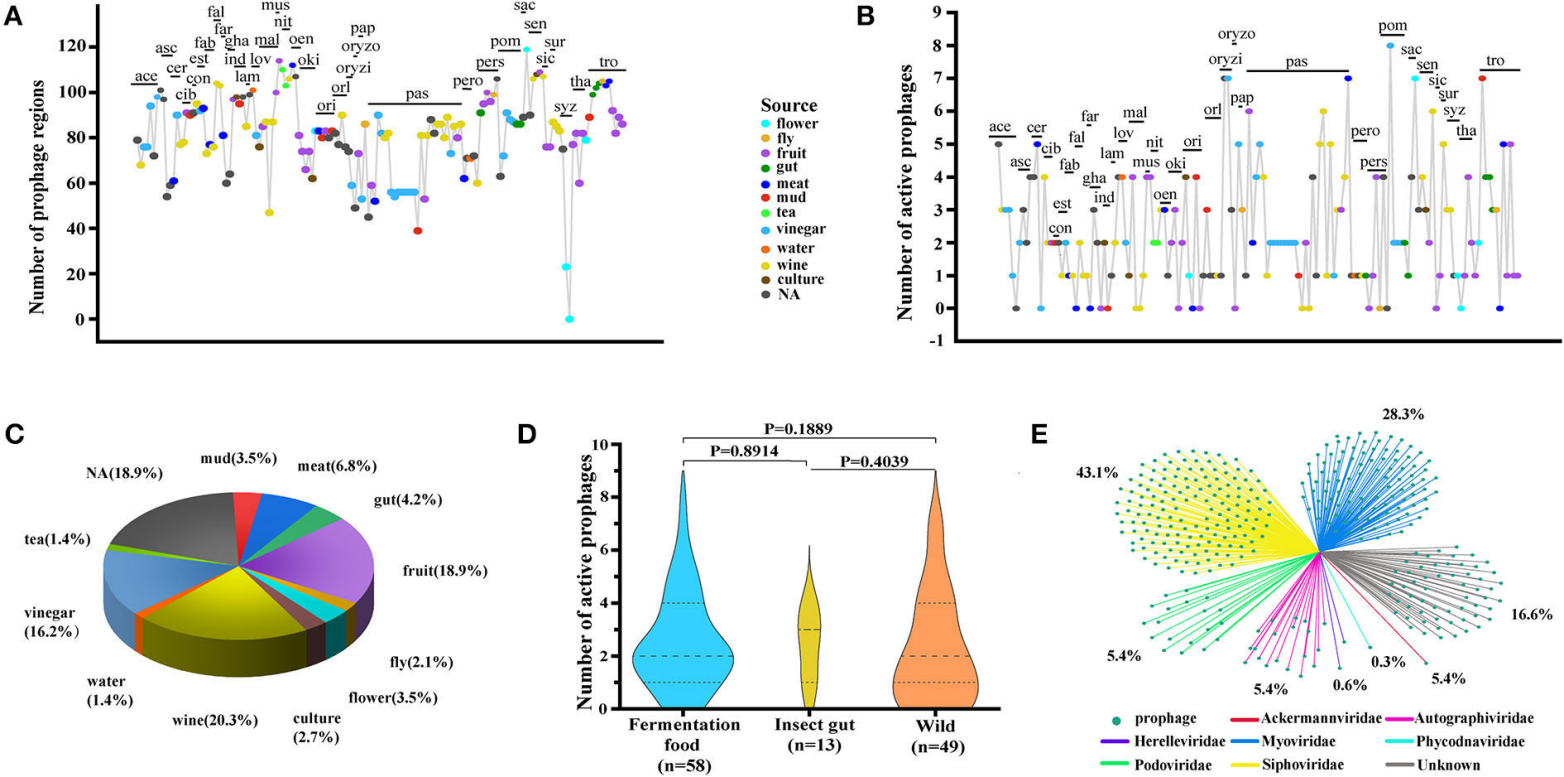
Depiction of prophages for 34 *Acetobacter* species. **(A)** All prophage fragments predicted in *Acetobacter*. **(B)** Active prophages predicted in *Acetobacter*. The above serial numbers are the classification of *Acetobacter* species (see Supplementary Table S1): *A. aceti* (ace); *A. ascendens* (asc); *A. cerevisiae* (cer); *A. cibinongensis* (cib); *A. conturbans* (con); *A. estunensis* (est); *A. fabarum* (fab); *A. fallax* (fal); *A. farinalis* (far); *A. ghanensis* (gha); *A. indonesiensis* (ind); *A. lambici* (lam); *A. lovaniensis* (lov); *A. malorum* (mal); *A. musti* (mus); *A. nitrogenifigens* (nit); *A. oeni* (oen); *A. okinawensis* (oki); *A. orientalis* (ori); *A. orleanensis* (orl); *A. oryzifermentans* (oryzi); A. oryzoeni (oryzo); *A. papaya* (pap); *A. pasteurianus* (pas); *A. peroxydans* (pero); *A. persici* (pers); *A. pomorum* (pom); *A. sacchari* (sac); *A. senegalensis* (sen); *A. sicerae* (sic); *A. suratthaniensis* (sur); *A. syzygii* (syz); *A. thailandicus* (tha); and *A. tropicalis* (tro). **(C)** Statistics of the source of *Acetobacter* isolates. **(D)** Comparison of predicted active prophages among the fermentation habitat group, the insect gut habitat group, and the variable habitat group. Statistical significance tests were performed using the nonparametric Mann–Whitney *U*-test, and two-tailed values of *p-*value were calculated. **(E)** Statistics of the family of prophages.

We performed a prophage search on the 148 genomes from 34 *Acetobacter* species and found 12,000 prophage fragments. Of which, most genomes contained more than 40 prophage fragments (Figure 1A). Specifically, *A. sacchari* TBRC 11175 (sac) isolated from flower showed the highest number of prophage fragments (119). Interestingly, no prophage fragments were predicted in the *A. syzygii* UBA5806 (syz) genome. Among the 12,000 prophage fragments, only 350 fragments were active and intact prophages. Moreover, 86.5% (128/148) of *Acetobacter* genomes harbored active prophages. The number of active prophages varied from 1 to 8 with a mean value of 2.7. Most *Acetobacter* strains contained 1–5 prophages, while a few strains like *A. oryzifermentans* SLV-7 (oryzi), *A. pomorum* SH (pom), and *A. sacchari* TBRC 11175 (sac) contained 7, 8, and 7 prophages, respectively (Figure 1B; Supplementary Table S1). The species *A. pasteurianus* is adopted universally by the food industry, and its active prophage number ranged from 0 to 7. The industrial species *A. pasteurianus* NBRC 3284 and *A. pasteurianus* Ab3 contained 1 and 5 active prophages, respectively, suggesting the evolutionary diversity of these strains. No active prophages were found in three species: *A. farinalis* (far), *A. papaya* (pap), and *A. suratthaniensis* (sur). These active prophages are embedded in the *Acetobacter* genome and are certainly expected to impact the host's physiological functions.

The fermented food biotopes like vinegar and wine production contribute to 16.2 % (24/148) and 20.3 % (30/148) distribution of *Acetobacter* strains recorded in NCBI genome data, respectively (Figure 1C; Supplementary Figure S1). Also, *Acetobacter* strains universally exist in variable habitats of flowers/fruits and relative stable habitats of the gut of flies (22.4 and 5.6 %, respectively). To investigate whether the number of active prophages of *Acetobacter* was related to the habitats, we divided 120 *Acetobacter* strains (except for 8 termed as NA) into fermented food habitat group (vinegar, wine, and tea, *n* = 58), insect gut habitat group (fly, gut, and culture, *n* = 13), and wild habitat group (flower, fruit, meat, mud, and water, *n* = 49) (Figure 1D). Unexpectedly, no significant difference in the number of prophages was observed among the three groups (*p* > 0.05). Nevertheless, we noticed that the strains possessing the active prophage counts of more than five mainly existed in the fermented food and wild habitat groups. For example, the aforementioned *A. pomorum* SH (pom) and *A. oryzifermentans* SLV-7 (oryzi) strains isolated from vinegar had seven and eight prophages, respectively, and *A. sacchari* TBRC 11175 (sac) strain isolated from flowers contained seven prophages. These findings suggested that a stressful and complex microbial community niche may prompt the integration of prophages into *Acetobacter* genome, which confer underlying advantages or disadvantages to the host evolution. Notably, except for the 16.6% of active prophages, the rest of the phages exclusively belonged to the order *Caudovirales* with reference to the NCBI virus database (Figure 1E). Of which, the families *Siphoviridae* and *Myroviridae* prophages yielded the higher distribution ratio of 43.1 and 28.3%, respectively, while the families *Herelleviridae* and *Phycodnaviridae* showed less distribution by 0.6 and 0.3%, respectively.

### Genomic characteristics of *Acetobacter* prophages

**Figure 2:**
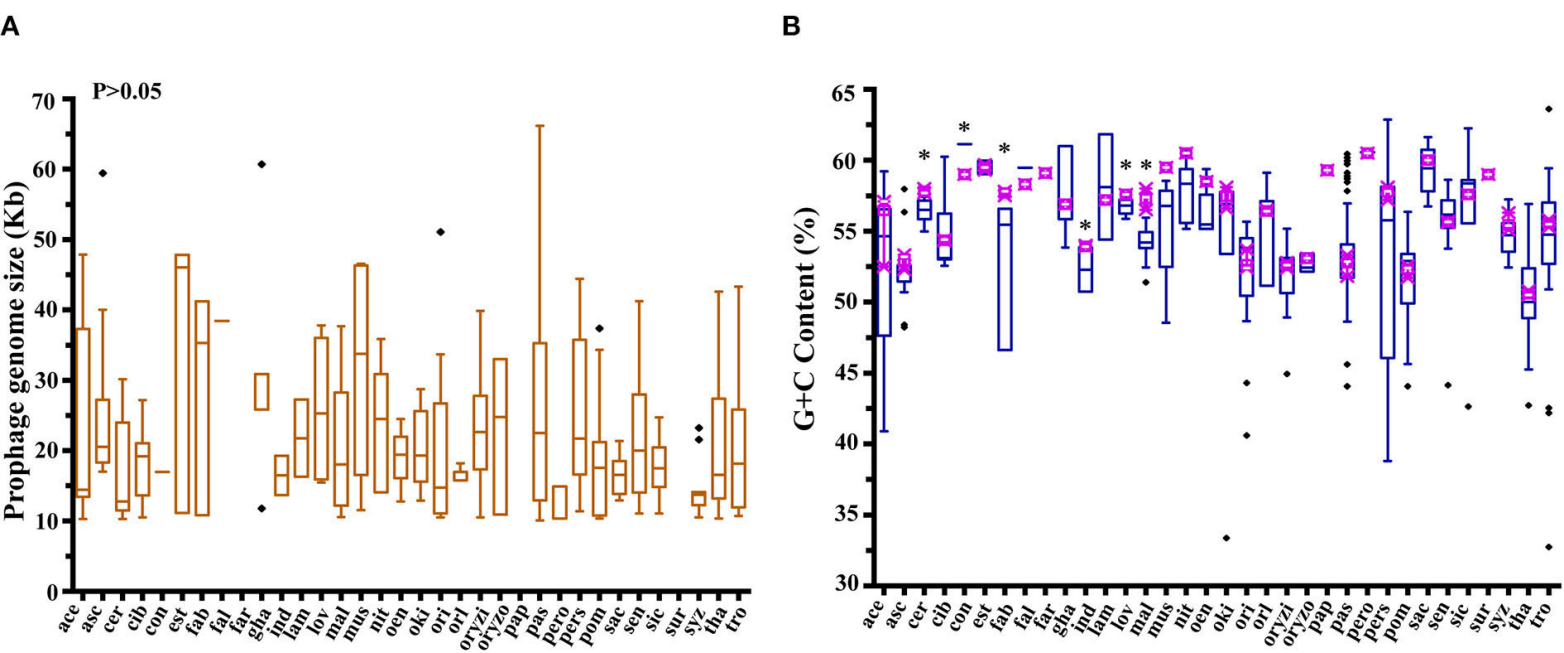
Characterization of 350 active prophage genomes. **(A)** Comparison of prophage genome size. A non-parametric test (Kruskal–Wallis) using multiple independent samples was performed. **(B)** Comparison of the GC content of the prophage genomes with their respective hosts. Blue boxes represent prophages and red boxes represent host strains. ♦, outliers. Non-parametric Mann–Whitney *U*-test and two-tailed method were used to check *p-*values comparing prophages and hosts in each group, *p < 0.05.

The size of 350 active prophages varied from 10.074 kb (phiBCRC14145-4) to 66.17 kb (phiAb3-5) with an average of 21.01 ± 7.98 kb (median ± interquartile range), and a significant difference was found at the intraspecies level. Interestingly, the size of the active prophage genome was exactly the span of the prophage genome size of *A. pasteurianus* (pas) (Figure 2A; Supplementary Table S2). In line with the prophage genome size of *A. pasteurianus*, the prophage sizes of *A. aceti* (ace), *A. estunensis* (est), and *A. musti* (mus) showed a similar trend. Prophage genomes from *A. ascendens* (asc), *A. ghanensis* (gha), *A. orientalis* (ori), *A. pomorum* (pom), and *A. syzygii* (syz) had high or low outliers. In addition, no significant differences in the active prophage genome sizes were observed at the host interspecies level.

The variation in GC content is closely related to mutation, selection, and recombination of microbial genes (Lassalle et al., 2015; Hellweger et al., 2018). It was found that the GC content of *Acetobacter* prophages ranged from 32.73% (phiNBRC 101654-2) to 63.62% (phiLMG 1663-2) at the interspecies level (Figure 2B; Supplementary Table S2). The phiNBRC 3277-4 and phiNBRC 3208-5 from *A. pasteurianus* (pas) NBRC 3277 and *A. pasteurianus* 3280, respectively, gave a low GC content of 44.07%, while phiBCRC 14145-1 from *A. pasteurianus* BCRC 14145 displayed a high GC content of 60.3%. The prophage genomes obtained from *A. aceti* (ace), *A. pasteurianus* (pas), *A. persici* (pers), and *A. tropicalis* (tro) exhibited a significant difference in the GC content. Interestingly, the prophages with the maximum and the minimum GC content appeared in the form of outliers in *A. tropicalis* (tro). Meanwhile, the GC content of prophage genomes also exhibited significant differences at the host intraspecies level, such as in *A. cerevisiae* (cer), *A. conturbans* (con), *A. fabarum* (fab), *A. indonesiensis* (ind), *A. lovaniensis* (lov), and *A. malorum* (mal) (*p* < 0.05). The prophages from *A. conturbans* showed a higher GC content of 61.13% than its host which showed 59%, while prophages from the other five species (*A. cerevisiae* (cer), *A. fabarum* (fab), *A. indonesiensis* (ind), *A. lovaniensis* (lov), and *A. malorum* (mal)) exhibited significantly lower GC content than their hosts (Supplementary Tables S1, S2). Nevertheless, the GC content of prophages was overall in line with their hosts, implying the biocompatibility of prophage–host and their co-evolution in the microbial community.

**Figure 3:**
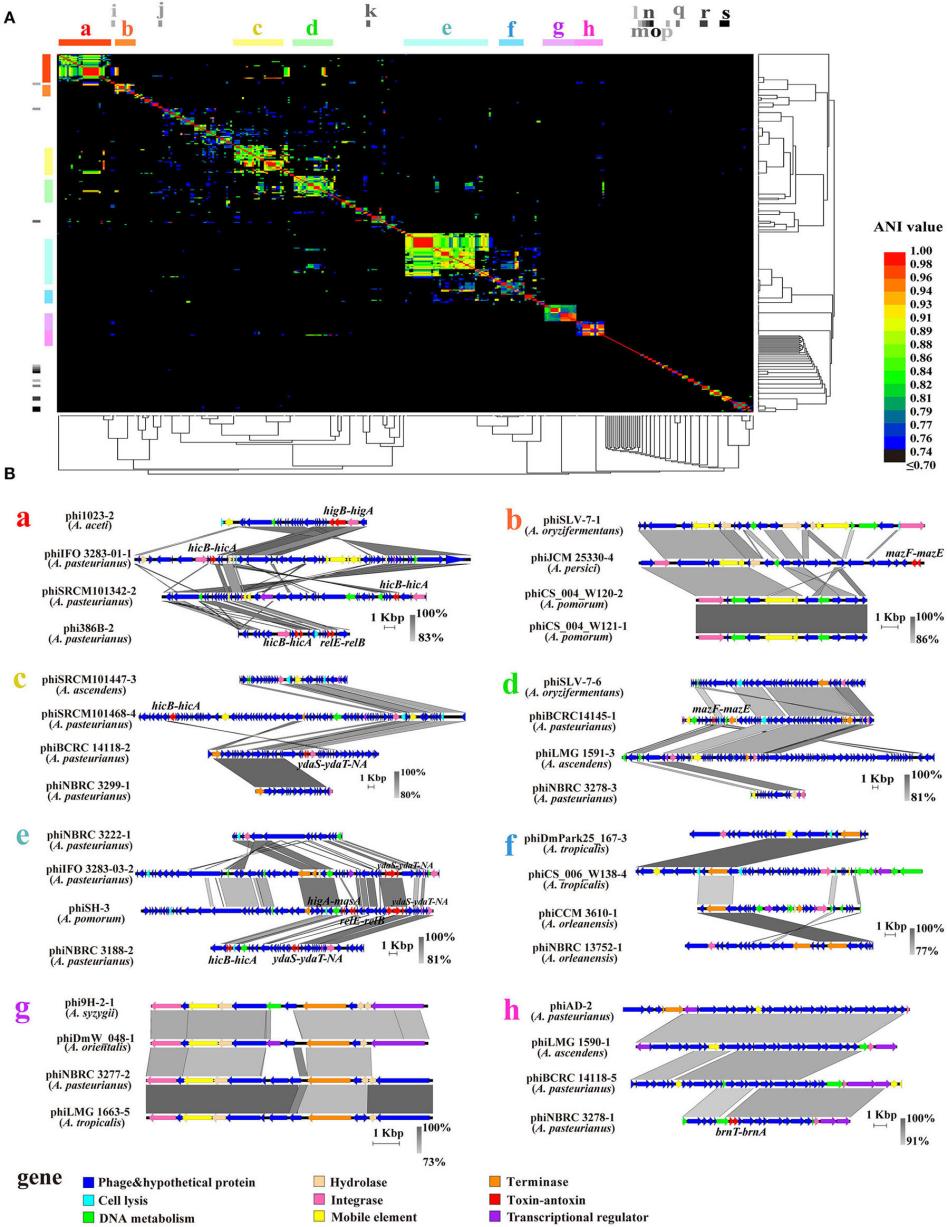
Analysis of 350 active prophage genomes. **(A)** The 350 prophage paired ANI values were clustered using a mean linkage hierarchical clustering method based on Pearson's distance for rows and columns in all clusters. The colors in the heat map represent different ANI values, gradually changing from blue (low identity) to red (high identity). a to h' represent the eight large subclusters and i to s' represent the small subclusters composed of prophages of a single Acetobacter species. The ANI values are shown in Supplementary Table S3. **(B)** Comparative genomics of prophages for each large subcluster 8 (a, b, c, d, e, f, g, and h). The gray area represents the similarity module and the similarity changes gradually from gray (low) to black (high).

In order to uncover the prophage diversity at the nucleotide level, we calculated the average nucleotide identity (ANI) for the 350 active prophage genomes (Supplementary Table S3). The ANI analysis results were then arranged in pairs in a matrix. Notably, only 5.01% (6,138/122,500) of the modules were observed with matching values higher than 70% (Supplementary Table S3), illustrating the low identity and high diversity among *Acetobacter* prophage genomes. Subsequently, the matrix was clustered by using the mean linkage hierarchical clustering method based on Pearson distance, and then visualized in the form of a heat map with a gradual change from low (black) to high identity (red) (Figure 3A). Exception of nineteen prophages with the ANI value less than 70%, the remaining 331 prophages could be grouped into 8 large subclusters (termed as a, b, c, d, e, f, g, and h, where each subcluster contained n > 10 prophages) and 42 small subclusters (each subcluster contained the prophages at 2 ≤ n ≤ 8, termed as i, j, k, …, and only the frontier i-s subclusters are labeled in the Figure 3A) based on the affinities. Specifically, the small subclusters i (*A. thailandicus*), j (*A. nitrogenifigens*), k (*A. lovaniensis*), l (*A. sacchari*), m (*A. nitrogenifigens*), n (*A. musti*), o (*A. fallax*), p (*A. sacchari*), q (*A. aceti*), r (*A. oeni*), and s (*A. thailandicus*) were predominantly composed of a single *Acetobacter* species, suggesting the host species are specific to the prophages. The remaining 31 small subclusters and 8 large subclusters of prophages divergently occurred in diverse *Acetobacter* species. Moreover, the prophages of each large subcluster were derived from different *Acetobacter* species with an average of 5.88 *Acetobacter* species per subcluster (Table 1). For example, the largest subcluster e contained 42 prophages derived from *A. aceti, A. pasteurianus, A. oryzifermentans, A. pomorum*, and *A. senegalensis*. The subclusters a and c contained 23 and 25 prophages from six and five host species, respectively. The prophages of subclusters b, f, and g were derived from seven different species each. There were many subclusters that contained only one prophage, implying their separated evolution. Generally, an ANI value above 0.95 suggests the same species (Jain et al., 2018). In the large subclusters, the phiSRCM 101447-2 from *A. ascendens* SRCM 101447 in subcluster c showed 95% identity with phiAb3-2 from *A. pasteurianus* Ab3 in subcluster a, and the phiCICC 22518-1 from *A. pasteurianus* CICC 22518 in subcluster c showed 98.1% identity to the phiSRCM 101447-1 from *A. ascendens* SRCM 101447 in the subcluster a. Similarly, the phiLMG 1590-3 (96.8% identity), phiLMG 1591-1 (96.8%), phiBCRC 14118-1 (97.4%), phiBCRC 14118-2 (96.1%), and phiLMG 1590-2 (95.8%) in the subcluster c showed high identity to phiSRCM 101447-1 from *A. ascendens* SRCM 101447 in the subcluster a. Specifically, though the phiUBA 5402-1 shared a high identity of 98.8% with phiUBA 5402-2 of *A. orientalis* UBA 5402, they were separately clustered into different subclusters according to the ANI analysis.

**Table 1:** Distribution of prophages grouped in the eight large subclusters.

**Subclusters**	**Total prophages**	**Prophages per host species**		**Host species**	**Host species classification**
		**min**	**max**		
a	23	1	17	6	*A. aceti*; *A. ascendens*; *A. orientalis*; *A. pasteurianus*; *A. persici*; *A. pomorum*
b	10	1	3	7	*A. aceti*; *A. malorum*; *A. oryzifermentans*; *A. persici*; *A. pomorum*; *A. senegalensis*; *A. tropicalis*
c	25	2	10	5	*A. ascendens*; *A. oryzifermentans*; *A. oryzoeni*; *A. pasteurianus*; *A. pomorum*
d	20	1	15	4	*A. ascendens*; *A. oryzifermentans*; *A. pasteurianus*; *A. pomorum*
e	42	1	27	5	*A. aceti*; *A. oryzifermentans*; *A. pasteurianus*; *A. pomorum*; *A. senegalensis*
f	12	1	4	7	*A. indonesiensis*; *A. malorum*; *A. orleanensis*; *A. pasteurianus*; *A. persici*; *A. pomorum*; *A. tropicalis*
g	17	1	5	7	*A. orientalis*; *A. oryzifermentans*; *A. oryzoeni*; *A. pasteurianus*; *A. pomorum*; *A. syzygii*; *A. tropicalis*
h	14	1	9	6	*A. ascendens*; *A. cibinongensis*; *A. orientalis*; *A. pasteurianus*; *A. senegalensis*; *A. tropicalis*

In addition to the ANI analysis, to further investigate whether the prophage functional gene structures in each subcluster were continuous or segregated, we packaged the functional annotations of the prophage genomes of the eight large subclusters and selected the dominant prophage types from each subcluster for visualization (Figure 3B). Accordingly, 32 representative prophages were selected and 12 kinds of proteins were figured out, including phage (capsid, head protein, tail protein, and other structural proteins), cell lysis, DNA metabolism, hydrolase, integrase, mobile element protein, terminase, type II TAs, transcriptional regulator, and hypothetical protein. The composition and architecture of the prophage genomes displayed a high diversity within each subcluster, except for the g and h subclusters where the ANI and arrangement of prophage genomes shared a high identity. In subcluster a, the integrase showed a low identity below 83% among the four prophages, and the hydrolase was only found in phiIFO 3283-01-1. Notably, TAs located in multiple gene loci did not show any consistency, e.g., nearby the integrase genes in subcluster a, the transcriptional factors or cell lysis genes in the subcluster e, and the DNA metabolism genes in the subcluster h. The subclusters f and g did not have any TAs. Additionally, although prophages were clustered based on the ANI analysis, the genome size in each subcluster varied greatly, which was in line with the results of the distribution of prophage biodiversity.

### Distribution of type II TAs in the *Acetobacter* prophages

**Figure 4:**
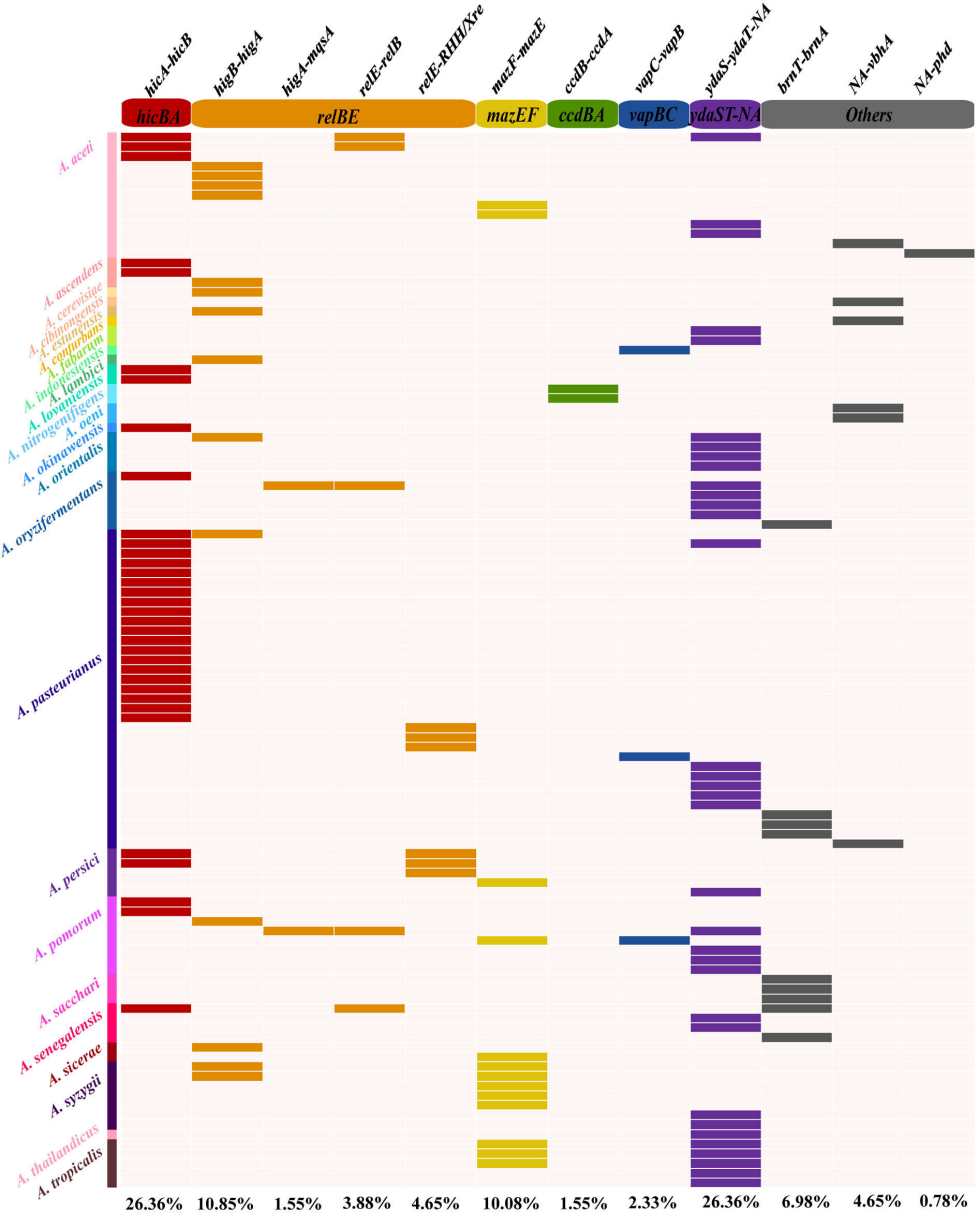
Distribution of type II TAs in *Acetobacter* prophages. Gene names and the corresponding type II TA families are displayed on the top of the heat map. Different colors on the left side represent different *Acetobacter* species.

Currently, TAs have been uncovered with a variety of physiological functions involved in multiple networks in the cellular metabolism and regulation process, specifically in the defense mechanisms and self-addictive evolution with the flanked genes (Kamruzzaman et al., 2021). Based on the TAfinder software analysis, 129 type II TA loci on 109 out of 350 active prophage genomes were predicted. Totally, 12 kinds of TAs were found, including *hicA-hicB, higB-higA, higA-mqsA, relE-relB, relE-RHH-Xre, mazF-mazE, ccdB-ccdA, vapC-vapB, ydaS-ydaR-NA, brnT-brnA*, NA*-vbhA*, and NA*-phd* (Figure 4; Supplementary Table S4). Moreover, only one copy of each type II TA existed in each prophage genome. The *hicA-hicB* showed an equally high distribution of 26.35% (34/129) in the nine *Acetobacter* species, while 34 *ydaS-ydaR-NA* was distributed more diversely in 12 species (Figure 4). *NA-phd* was found to exist only in phiDSM3508-1 of *A. aceti*. Similarly, the *ccdB-ccdA* was found in two prophages (phiDSM 23921-2 and phiNBRC 105050-2 of *A. nitrogenifigens*). Additionally, most prophage genomes (115/129) contained just one kind of TAs, while the phiJCM 20276-3 of *A. aceti*, phidm-6 of *A. oryzifermentans*, phiSH-3 of *A. pomorum*, and phi108B-2 of *A. senegalensis* contained three different TAs, respectively.

*Acetobacter pasteurianus* showed a relatively high abundance of TAs in their prophage genomes (Figure 4). Thirty-three out of eighty-nine prophage genomes contained TAs with a yield of 37.09%, consisting of the dominant species *hicB-hicA* (20/33), *ydaS-ydaT-NA* (5/33), *brnT-brnA* (3/33), and *relE-RHH/Xre* (3/33). *A. aceti* is another dominant species in fermented wine and vinegar, and 54.54% of its prophage genomes (12/22) contained TAs, and the dominant TAs included *higB-higA, hicA-hicB*, and *relE-relB*. The species *A. syzygii* can usually be isolated from wine and flower, and 7 out of the 13 prophage genomes had TA loci, such as *higB-higA, mazF-mazE*, and *ydaS-ydaT-NA*. A similar abundance of TAs existed in the prophage genomes of other *Acetobacter* species, including *A. oryzifermentans* (42.86%, 6/14), *A. persici* (45.45%, 5/11), *A. pomorum* (38.10%, 8/21), *A. sacchari* (42.86%, 3/7), *A. nitrogenifigens* (50%, 2/4), and *A. fabarum* (66.67%, 2/3). The loci of these type II TAs mainly belonged to six classical two-component families (*hicBA, relBE, mazEF, ccdBA, vapBC*, and *brnTA*), one three-component family *paaR*-*paaA*-*parE*, and other unknown families. The distribution and abundance of TAs harbored by the prophages appear to be strain-specific, which was consistent to the extensive transmission across the species of the prophage.

### CRISPR-Cas system existence in *Acetobacter*

**Figure 5:**
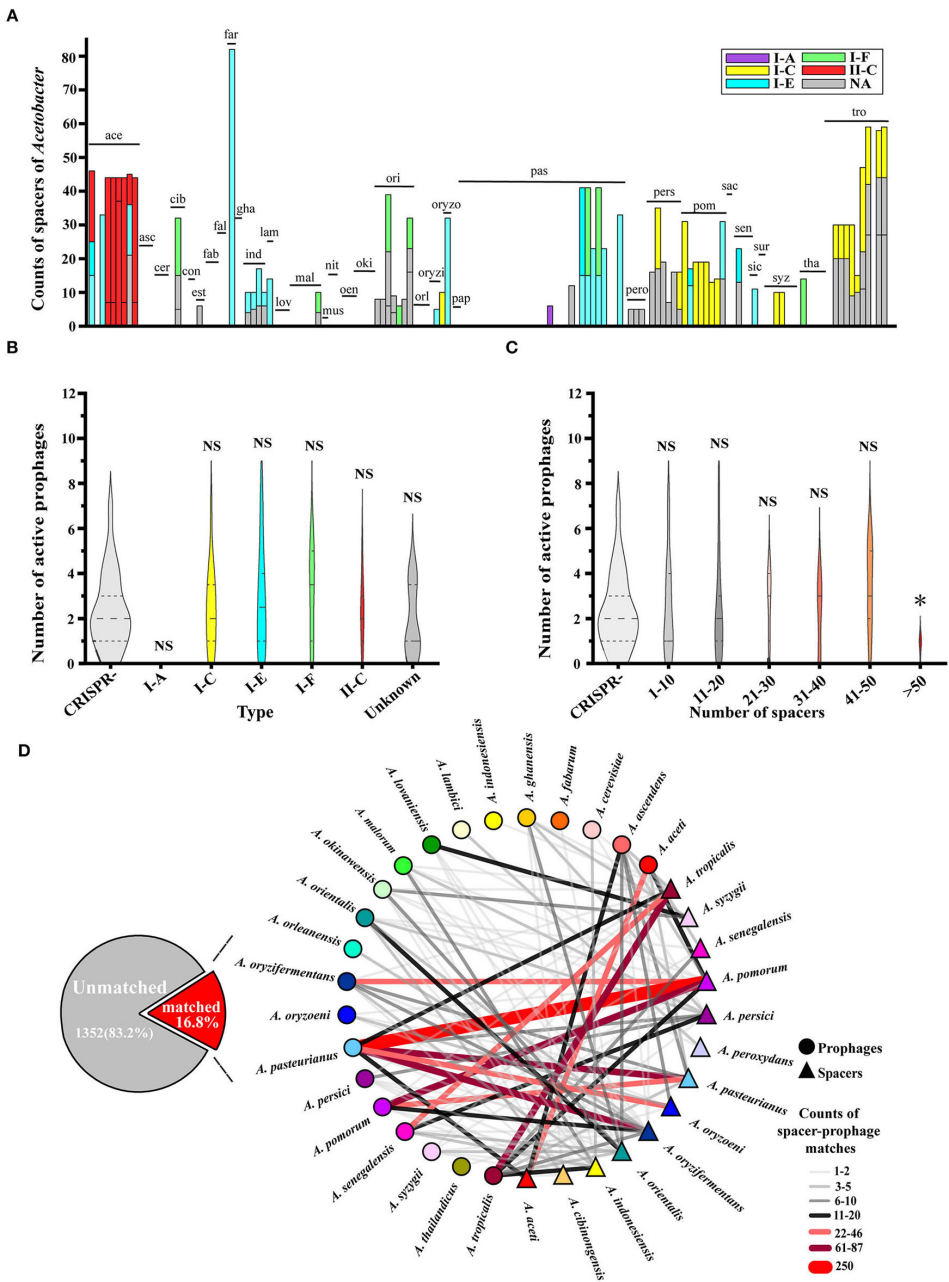
Analysis of CRISPR-Cas system spacers on the *Acetobacter* genome sequences. **(A)** The number of CRISPR-Cas system spacers counted for each *Acetobacter* species, and different colors represent different types of CRISPR-Cas systems. Purple I-A, yellow I-C, blue I-E, green I-F, red II-C, and black NA. **(B)** Relationship between CRISPR-Cas system types and prophage numbers. **(C)** Relationship between the genomic CRISPR-Cas system spacers and the number of prophages in *Acetobacter*. X-axis is the total number of CRISPR-Cas system spacers, and Y-axis is the number of prophages. Non-parametric Mann–Whitney U-test and two-tailed method were used to calculate *p-*values. **(D)** Spacer-prophage matching network.

Even though *Acetobacter* has some interest in the co-existence with prophages, the host itself possesses the corresponding resistance systems to prevent the risk of prophage intrusion that may further limit the growth or cause a serious burden to hosts (Doron et al., 2018; Owen et al., 2021). Among the driver mechanisms responsible for bacterial defense against phage invasion, the CRISPR-Cas system is regarded as one of the classical approaches (Horvath and Barrangou, 2010). Based on the spacer sequences, we scanned the *Acetobacter* genomes available currently. Of which, 44.6% (66/148) of *Acetobacter* genomes possessed CRISPR-Cas systems, and 23% had 2-3 loci. The main CRISPR-Cas systems in the *Acetobacter* belonged to the type I-A, I-C, I-E, I-F, and type II-C, respectively (Figure 5A; Supplementary Table S5). Specifically, we observed that type II-C CRISPR-Cas system existed only in *A. aceti* (ace) and type I-A only in *A. pasteurianus* UBA5418 compared to the prevalence of type I-E in the *Acetobacter* species. Additionally, the mean count of spacers of *Acetobacter* genome was up to 11 sequences. Thirty-five *Acetobacter* genomes harbored 43 putative or identified CRISPR-Cas arrays, and 82 genomes seemed not to contain any spacers or CRISPR-Cas systems, indicating the uneven distribution of CRISPR-Cas systems in *Acetobacter* (Supplementary Table S5). Specifically, *A. farinalis* LMG26772 (far) genome contained the highest spacer counts up to 82. However, 4 ambiguous and 77 inactive prophages were detected on its genome without any active prophages. Therefore, we determined the relationship between the active prophage number and their host's CRISPR-Cas systems (Figure 5B). No significant difference in the number of active prophages was demonstrated among each CRISPR-Cas type and the unknown groups (*p* > 0.05), while a similar phenomenon was observed for the number of prophages against the number of spacers in 1–10, 11–20, 21–30, 31–40, and 41–50 groups, respectively (*p* > 0.05). But when the host genome contained more than 50 spacers, the prophage numbers significantly reduced to <2 (*p* < 0.05) (Figure 5C).

When a total of 1,625 predicted spacers in the 148 *Acetobacter* genomes were mapped to all the active prophage genomes, only16.80% of the spacers were matched with 95% identity (Figure 5D), suggesting that most of the prophages were not recorded by their hosts. The spacers from 13 *Acetobacter* species presented the intricate information network with the prophages from 21 *Acetobacter* species (Supplementary Table S6). One host species contained spacers pairing with a few different prophages, while one prophage may infect multiple different hosts. Particularly, the spacers of *A. pomorum* matched with the most number of prophages from 12 *Acetobacter* species, and exhibited the highest number of prophage matching in *A. pasteurianus*. For instance, the match counts between the prophages of *A. pasteurianus* and the spacers of *A. pomorum* were found to be 250, between *A. pasteurianus* and *A. oryzifermentans* werein the range of 61- 87, between *A. pasteurianus* and *A. oryzoeni* were in the range of 22–46, and between *A. tropicalis* and *A. aceti* were in the range of 11–20. However, the match counts between the spacers of *A. pasteurianus* and the prophages of *A. pasteurianus* or *A. pomorum* ranged from 22 to 87, which is less than the abovementioned values. From the prophage standpoint, the prophages from *A. pasteurianus* could find much more counter-spacers from a variety of *Acetobacter* species. Therefore, the species matching analysis and ANI analysis together constructed a global model of host strain specificity to *Acetobacter* prophages.

**Figure 6:**
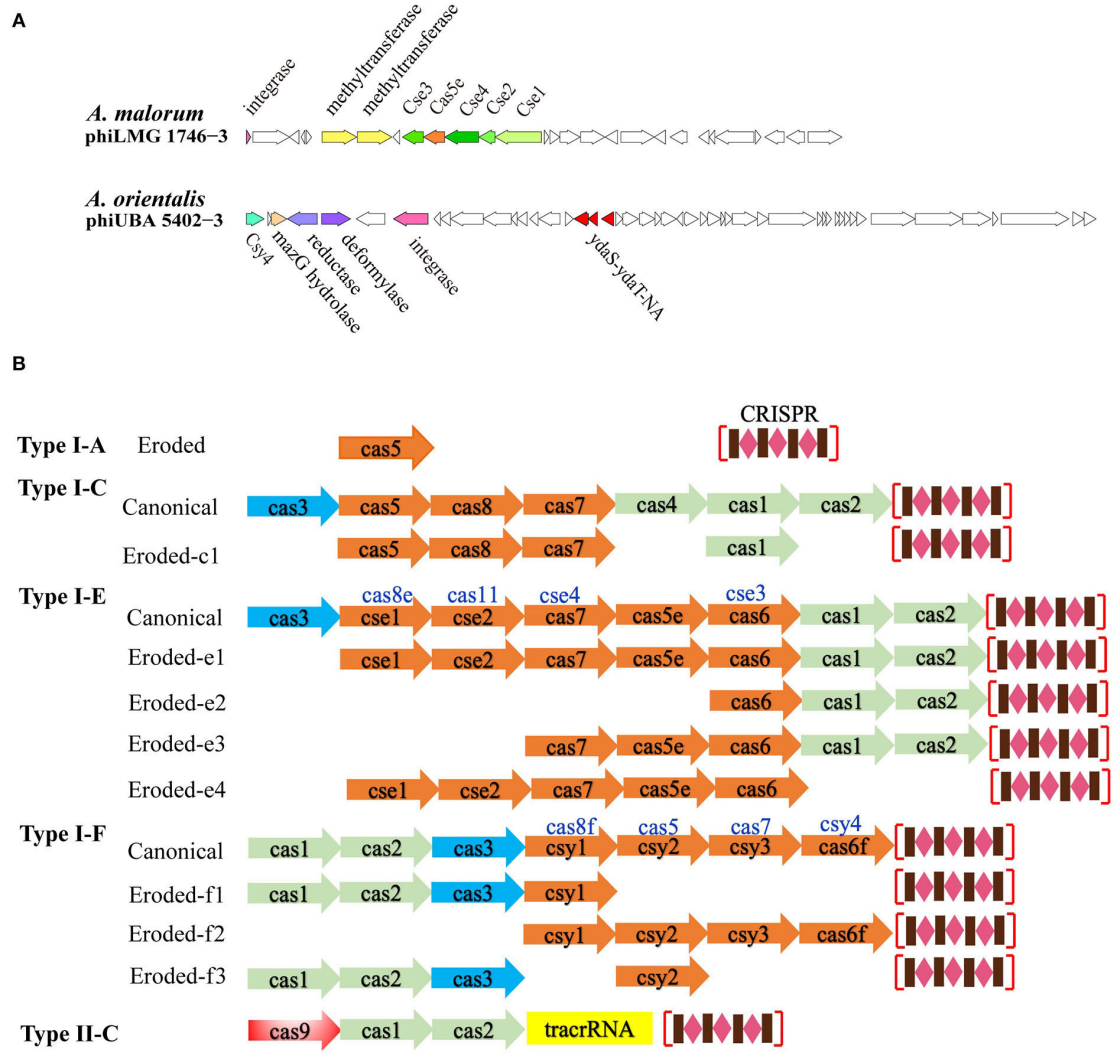
Architecture of prophage genomic loci for the subtypes of CRISPR-Cas systems. **(A)** Two unique genomes of the *Acetobacter*-specific prophages harbored the eroded type I-E and I-F CRISPR-Cas loci. **(B)** Diagrams of the intact or incomplete CRISPR-Cas loci representatives existed on *Acetobacter* genomes. Genes encoding the adaption, effector, and target cleavage module are colored in green, orange, and blue, respectively.

Specifically, several genes encoding the CRISPR-related proteins were found, including *cse1, cse2, cse3, cse4*, and *cas5e* in the prophage phiLMG 1746-3 (hosted by *A. malorum* LMG 1746) and *csy4* in the prophage phiUBA 5402-3 (hosted by *A. orientalis* UBA 5402) (Figure 6A). Also, nine spacers were detected on phiUBA 5402-3 genome, while none were found on phiLMG 1746-3 (Supplementary Table S7). The phiLMG 1746-3 showed 87% similarity to *Enterobacteria* phage-BP-4795 (temperate, *Siphoviridae*, GenBank accession number AJ556162) which consisted of 85 ORFs, including 2 IS 629 elements and 3 morons (Creuzburg et al., 2005). The host *A. malorum* LMG 1746 was isolated from fruit and predicted to harbor four active prophages and a total of 100 prophage fragments. Two CRISPR-Cas loci were found on the bacterial genome, where one was an eroded type I-F structure composed of *cas6, csy1, csy2*, and *csy3*, while the other loci only included *cas1, cas2*, and *cas3* (Figure 6B). The phiUBA5402-3 showed a 90% similarity with *Brucella* phage BK (lytic, *Podoviridae*, GenBank accession number KC556893) (Farlow et al., 2014). The host *A. orientalis* UBA 5402 was isolated from mud and harbored 3 active prophages and 83 prophage fragments, and three CRISPR-Cas system modules occurred on the bacterial genome, i.e., one intact type I-F CRISPR-Cas system and two unknown fragments which just contained adaption modules *cas1* and *cas2* or orphan effector element *cas3*, respectively (Figure 6B; Supplementary Table S5). In comparison to the canonical modular organization of CRISPR-Cas systems, *cse1- 4* and *cas5e* usually exist in the Cse subtype I-E, while *csy4* does in the Csy subtype I-F. Meanwhile, lots of the truncated CRISPR-Cas modules that reside on the *Acetobacter* genomes, as well as those unidentified CRISPR-Cas loci, remained either adaption elements of *cas1*, cas2, *cas4*, and effector *cas3* or kinds of combination.

## Discussion

*Acetobacter* is a predominant genus that occurs widely in a variety of artificial or wild environmental ecosystems, particularly several species, such as *A. pasteurianus*, are historically adopted for industry-of-interest. Since the first phage A-1 of *Acetobacter suboxydans* ATCC 621 (now reclassified as *Gluconobacter oxydans*) was reported in 1979 (Schocher et al., 1979), only five *Acetobacter*-specific phages (MO 1, Acm 1, phiAP1, phiAO1, and phiAX1) have been identified till 2021. Currently, a large amount of genomic data of *Acetobacter* is available on NCBI, which enables the *in silico* investigation of diversity, genome features, and ecological functions of *Acetobacter*-specific prophages and their potential relationship with hosts.

In parallel to the distribution of *Acetobacter* species, which shows high diversity in complexity and habitats in nature (De Roos et al., 2018), the 350 predicted active *Acetobacter*-specific prophages exhibited a similar diverse distribution and abundance at the levels of host species and strain. About 86.5% (128/148) of *Acetobacter* strains presented active prophages, and similar distribution yields were found in *Lactobacillus* (64.1%), *Pseudomonas* (46.7%), and *Klebsiella* (69.3%) (Marques et al., 2021; Pei et al., 2021; Johnson et al., 2022). Generally, increased environmental stresses may stimulate a bacterium to evolve a series of mechanisms against harsh environments. In this process, prophages are usually supposed to confer the host some advantages in competition. However, we did not find a significant difference between the prophage abundance and distribution specific to the habitat complexity of their *Acetobacter* hosts. This finding is not consistent with the investigation of the intact *Lactobacillus* prophages, where the prophage counts harbored by their host strains obtained from the fermented food group definitely displayed a difference from that obtained from the human/mammal group, i.e., a habit-associated pattern is observed (Pei et al., 2021). A more complex community may confer the host more options for co-survival with mobile genetic factors, including phages like that in *Lactococcus* (Matsutani et al., 2020). The investigation of 120 distinct phages from 303 *Lactobacillus helveticus* strains of natural whey starter cultures revealed that the high incidence of phage contamination cases can be recovered by high biodiversity of bacterial hosts and consequently maintaining a successful acidification capacity (Mancini et al., 2021). *A. pasteurianus* strains are adopted universally by food industries, while the active prophage numbers ranged from 0 to 7 whenever it occurs in wild or fermented foods, suggesting similar survival to the enteral genetic element challenges during the evolution process. Considering the fact that *Acetobacter* strains isolated from either industrial scenes or fly guts exactly and initially come from wild environmental niches and limited artificial domestications occur even for vinegar and wine production, we preferably suppose a unique strain-specific distribution for *Acetobacter*-specific prophages.

Also, the genome size, composition, and architecture of *Acetobacter*-specific prophages displayed a significant difference at the strain level but not at the species level of the host. A total of 350 active prophages exhibited the genome size varying from 10.074 to 66.17 kb with a significant difference, while a similar difference was not found at the species level. Furthermore, we observed that 32.73–63.62% GC content of prophages matched with that of their host *Acetobacter* strains (Figure 2B), highlighting the strong connection between a prophage and its cognate host strain. This observation was supported further by the ANI analysis, which exhibited a generally low identity and high diversity among *Acetobacter* prophage genomes (Figure 3A). Each large subcluster contained prophages derived from multiple *Acetobacter* species, suggesting that prophages can spread broadly across species. The same phenomenon was observed in *Lactobacillus* and *Oenococcus* prophages (Claisse et al., 2021; Pei et al., 2021; Qin et al., 2022). Based on the aforementioned findings, we conclude that most of the *Acetobacter*-specific prophages are strain-specific and can transmit across both intra- and interspecies levels. The co-evolution of prophage with its host bacterium often occurs at the strain level, which is in line with the significant difference observed in the functional gene organization of *Acetobacter*-specific prophages (Figure 3B). Bacterial genes may integrate into the prophage genome during replication and result in a longer genome or lose redundant genes from the prophage in order to facilitate its own replication, which results in the intermingling of host and prophage gene fragments and then changes the genome size of prophages from host species. This biodiversity in prophage genomes increases the host population-level diversity and drives the continuous host evolution (Nawel et al., 2022). It is rational that similar ecological functions can be mirrored by the *Acetobacter*-specific prophages, and the rich diversity and broad diffusivity of *Acetobacter* prophage populations drive the genetic plasticity of *Acetobacter*.

The arms race between phage and bacteria promotes co-evolution. Lysogeny offers great benefit through the auxiliary functional genes in the prophage genome that always confer additional immunity roles against phage infection by multiple mechanisms (Jurenas et al., 2022). TAs from plasmids and bacterial genomes have been extensively investigated with regard to their multiple biological functions. Recently, their distribution and role in (pro)phages have become an attractive topic. However, only a few TAs on prophages have been identified (Jurenas et al., 2022). Previously, we have investigated the chromosomal *hicB-hicA* in *A. pasteurianus* Ab3 and annotated its functions (Xia et al., 2019, 2021a,b). The *hicB-hicA* was found on the phage phiS10 of *Streptococcus suis* and suggested a mechanism for the maintenance of the temperate phage (Tang et al., 2013). Considering the *hicB-hicA* ubiquity in the *Acetobacter* prophages, their roles remain to be investigated. The *ydaS-ydaT-NA* belongs to the family *paaR2-paarA2-parE2*; the latter occurs in CP933P prophage of *E. coli* O157:H7 and is regarded to stabilize this prophage (Jurenas et al., 2021). Notably, TAs of *higB-higA* and *hicB-hicA* are near integrase genes on prophage genomes (Figure 3B), and we speculate that the addiction of TAs benefits the stability of the latter, as what has been uncovered in *creT-creA* safeguarding *cas6* (Li et al., 2021). Till this study was presented, none of the *Acetobacter*-specific prophage-derived TAs has yet been identified, and the underlying functions, such as additional protection, host defense system, and lytic-to-lysogenic switch, deserve a comprehensive deciphering into the host–phage combat context. Whether the prophage-derived TA pair exerts a similar biological function as its counterpart derived from *Acetobacte*r chromosome and the plasmid is one open question.

Different from the bacterial defense mechanism at the population level by TAs acting in the Abi pattern in nature, the most intuitive patterns of phage defense should be CRISPR-Cas or restriction-modification systems at the single-cell level (Mohanraju et al., 2022). Our finding was in parallel to the previous study that the number of active prophages was necessarily neither correlated with CRISPR-Cas type number nor spacers number in the CRISPR arrays (Touchon et al., 2016). Considering that the *Acetobacter* species are not typical bacteria and efficient genetic tools are still lacking, our finding will benefit the development of novel CRISPR-Cas tools specific to *Acetobacter*. In this study, the type II-C CRISPR-Cas system was only found in *A. aceti*. The majority of *Acetobacter* genomes harbor either eroded unidentified CRISPR-Cas loci or no CRISPR-Cas loci (Supplementary Table S5), and in such cases, complex mechanisms led to the gradual elimination of a part or complete elements in the genome (Koonin and Makarova, 2017). The residual CRISPR modules varied in the composition of the adaptation, expression, or interference elements (Figure 6B), suggesting that each genome co-evolves differently in the same ecosystem and responds to divergent stress conditions. *Acetobacter*-specific type I-E organization seems a variant of the Cse type I-E derived from *Streptococcus thermophilus* DGCC7710, where *cse4* and *cas6* were replaced by *cas7* and *cas6*, respectively (Carte et al., 2014). The remaining four I-E loci occurred in the eroded organization for *Acetobacter* characterized without *cas3* interference function. Similarly, the *Acetobacter*-specific type I-F structure resembles the Csy subtype I-F derived from *Yersinia pestis* and *Pseudomonas aeruginosa* (Haft et al., 2005; Haurwitz et al., 2010). Intriguingly, we also observed that two specific prophages, phiLMG 1746-3 and phiUBA 5402-3, harbored the eroded genes encoding Cse1, Cse2, Cse3, Cse4, Cas5e, and Csy4, respectively (Figure 6A). The initial CRISPR repeat cleavage can be catalyzed by Cse3 (Cas6e) in the Cse type I-E and the Csy4 (Cas6f) of ‘Cas6 superfamily' in the Csy type I-F system, and both Cse3 and Csy4 recognize and cleave downstream of a stem-loop structure in associated CRISPR repeats while retaining the repeat stem-loop at the 3'-end of the Cse type I-E and Csy type I-F crRNAs (Carte et al., 2014). Therefore, the prophage phiLMG 1746-3 appeared to retain the minor eroded expression elements (*cse1-cse2-cse3-cse4-cas5e*) to process pre-crRNAs and release crRNAs, while the phiLMG 1746-3 might retain the recognition and cleavage ability of pre-crRNAs, which may be employed to cope with host defense and phage–phage competition. Currently, studies of viromes from various environmental ecosystems indicate that more (pro)phages possess CRISPR arrays and/or *cas* genes (Mohanraju et al., 2022). Considering the bi-directional evolutionary connection between CRISPR arrays and mobile genetic elements, the uncovering of type I-F elements-contained Tn7-like transposons or virus as well as the Cas4-contained phages is growing (Faure et al., 2019). Consistently, a type I-F loci with adaptation and CRISPR array was inserted into the ICP1-related phages infecting *Vibrio cholerae* (Seed et al., 2013). Therefore, we hypothesize that the eroded type I-E CRISPR array in the phiLMG 1746-3 genome and Cys4 type I-F locus in the phiUBA 5402-3 may reversely be captured from the Cse type I-E and Csy type I-F CRISPR-Cas loci on the bacterial genomes, respectively. Specifically, it is notable that the eroded type I-E CRISPR-Cas loci on the phiLMG 1746-3 genome are greatly distinct from its host bacterial genome which harbors the type I-F CRISPR-Cas system, indicating the different evolutionary pathways between the prophage and its host CRISPR-Cas system. Against the context of a long-term complex phage–host arm races, it is rational that prophages can promise a universal of additional defense mechanisms that are yet to be uncovered.

In summary, this study displayed some cues to elucidate the ecological, genetic diversity, and evolutionary synergy of prophages and their host *Acetobacter*. The *Acetobacter*-specific prophages exhibited a significant difference in distribution, abundance, and genetic biodiversity at the strain level but not at the species level, and few prophages have been identified and annotated. In parallel to the existence of an intact or incomplete CRISPR-Cas system in the host genome dominated by type I, a unique prophage harboring the eroded Cse type I- E CRISPR-Cas array implies its different evolution. In addition to 350 active prophages, more than 11,650 putative predicted intact prophage fragments were not sorted out here, and the function of these phage fossils is still an open question. A comprehensive experimental study combined with *in silico* comparative genomics will promote the understanding of the relationship between prophages and their *Acetobacter* hosts, and then afford the advantages to the fermented food industries in the future.

## Data availability statement

The original contributions presented in the study are included in the article/Supplementary material, further inquiries can be directed to the corresponding authors.

## Author contributions

CQ and JM designed and carried out the investigation, drafted the manuscript, and visualized the results. JL took part in the investigation. XL provided the general concept, supervised the work, and the writing of the manuscript. LZ provided the concept and reviewed the manuscript. All authors took part in the discussions and approved the final version.

## Funding

This work was funded by the Natural Science Foundation of Zhejiang Province (LY19C200002) to XL.

## Conflict of interest

The authors declare that the research was conducted in the absence of any commercial or financial relationships that could be construed as a potential conflict of interest.

## Publisher's note

All claims expressed in this article are solely those of the authors and do not necessarily represent those of their affiliated organizations, or those of the publisher, the editors and the reviewers. Any product that may be evaluated in this article, or claim that may be made by its manufacturer, is not guaranteed or endorsed by the publisher.
