# Supplementary material for: Comprehensive deciphering prophages in genus Acetobacter on the ecology, genomic features, toxin–antitoxin system, and linkage with CRISPR-Cas system
Source: Front Microbiol. 2022 Aug 2;13:951030. doi: 10.3389/fmicb.2022.951030 (PMC9379143; doi:10.3389/fmicb.2022.951030)
Supplement: Supplementary file 1 [file Data_Sheet_1.docx]

Supplementary Material

# Supplementary Figures


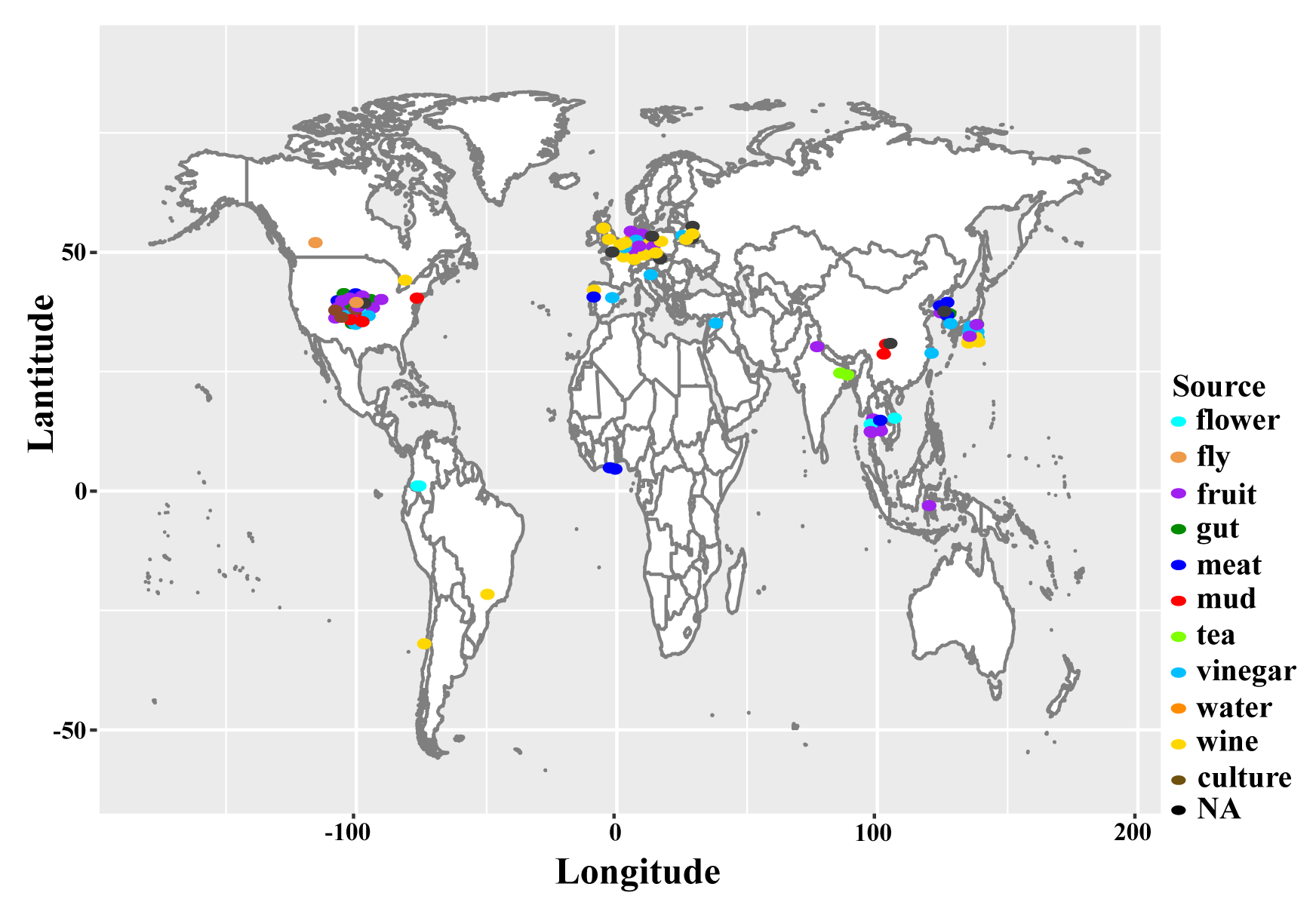


**Supplementary Figure 1.** Geographical distribution of 148 strains of 34 *Acetobacter* species. Different colors represent different biological sources, cyan: flower, brown: fly, purple: fruit, dark

green: gut, dark blue: meat, red: mud, light green: tea, blue: vinegar, orange: water, yellow: wine, brown, culture: black: NA.


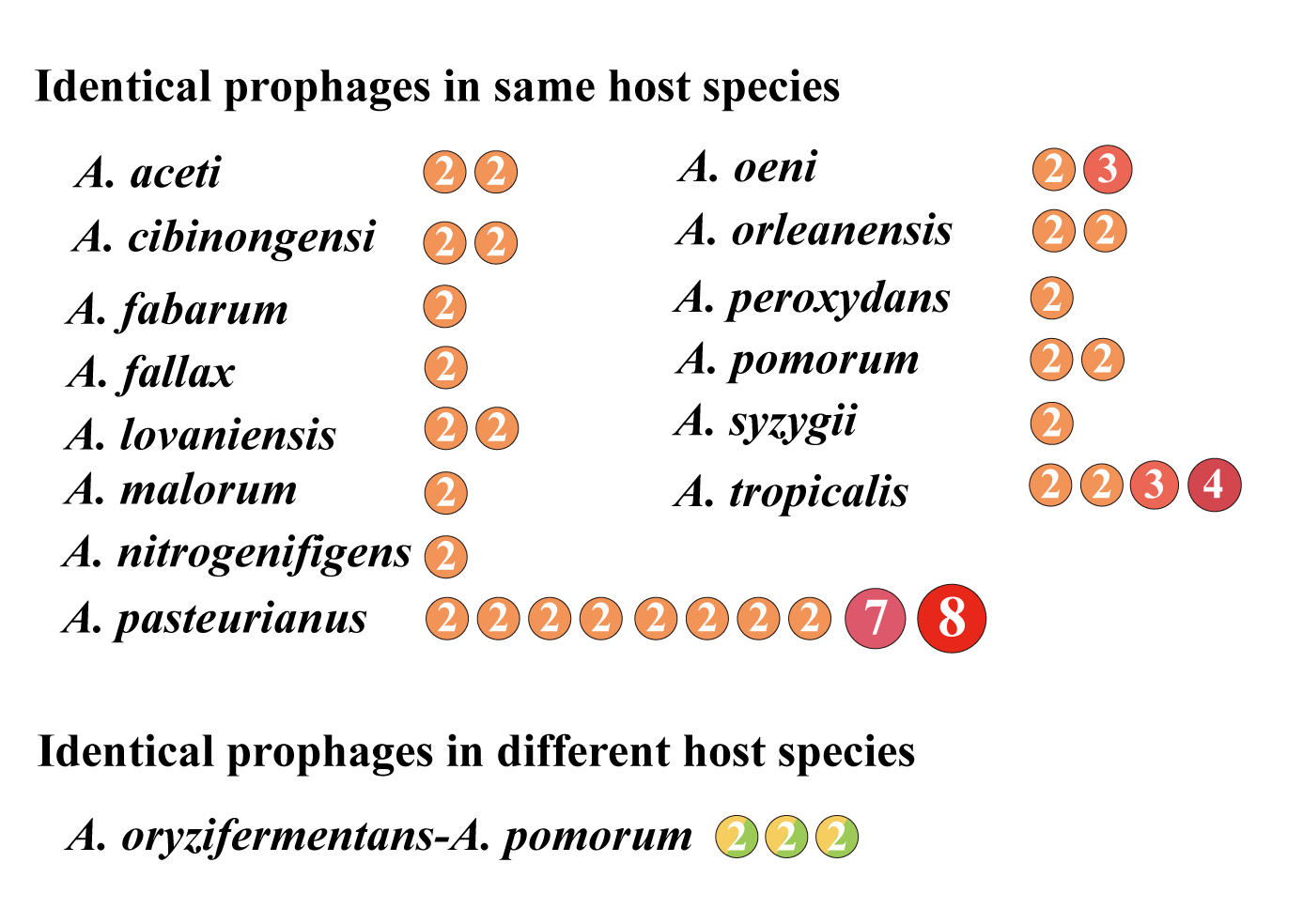


**Supplementary Figure 2.** Distribution of prophages with 100% sequence similarity. The circle represents the presence of identical prophages and the digit in the circle represents the total of the identical prophages.

# Supplementary Tables

**Table S1** The complete genome data *Acetobacter* strains available from NCBI isolated from variety of ecosystems around the world and predicted prophages amounts

**Table S2** The genetic and classification data for the predicted active prophages of *Acetobacter*

**Table S3** The data for ANI analysis of the active *Acetobacter*-specific prophages

**Table S4** The data for distribution and type of toxin-antitoxin systems on the prophage genomes

**Table S5** The CRISPR-Cas array data for the bacterial *Acetobacter*

**Table S6** The spacers prediction data for prophages and their host *Acetobacter* genomes

**Table S7** The spacer sequences on the prophage phiUBA5402-3
